# Joint Risk of Rainfall and Storm Surges during Typhoons in a Coastal City of Haidian Island, China

**DOI:** 10.3390/ijerph15071377

**Published:** 2018-06-30

**Authors:** Hongshi Xu, Kui Xu, Lingling Bin, Jijian Lian, Chao Ma

**Affiliations:** State Key Laboratory of Hydraulic Engineering Simulation and Safety, School of Civil Engineering, Tianjin University, Tianjin 300350, China; xuhongs@tju.edu.cn (H.X.); xk_tju@tju.edu.cn (K.X.); fj_np@126.com (J.L.); mac_tju@126.com (C.M.)

**Keywords:** typhoon, rainfall, storm surge, multivariate analysis, flood risk model

## Abstract

Public health risks from urban floods are a global concern. A typhoon is a devastating natural hazard that is often accompanied by heavy rainfall and high storm surges and causes serious floods in coastal cities. Affected by the same meteorological systems, typhoons, rainfall, and storm surges are three variables with significant correlations. In the study, the joint risk of rainfall and storm surges during typhoons was investigated based on principal component analysis, copula-based probability analysis, urban flood inundation model, and flood risk model methods. First, a typhoon was characterized by principal component analysis, integrating the maximum sustained wind (MSW), center pressure, and distance between the typhoon center and the study area. Following this, the Gumbel copula was selected as the best-fit copula function for the joint probability distribution of typhoons, rainfall, and storm surges. Finally, the impact of typhoons on the joint risk of rainfall and storm surges was investigated. The results indicate the following: (1) Typhoons can be well quantified by the principal component analysis method. (2) Ignoring the dependence between these flood drivers can inappropriately underestimate the flood risk in coastal regions. (3) The co-occurrence probability of rainfall and storm surges increases by at least 200% during typhoons. Therefore, coastal urban flood management should pay more attention to the joint impact of rainfall and storm surges on flood risk when a typhoon has occurred. (4) The expected annual damage is 0.82 million dollars when there is no typhoon, and it rises to 3.27 million dollars when typhoons have occurred. This indicates that typhoons greatly increase the flood risk in coastal zones. The obtained results may provide a scientific basis for urban flood risk assessment and management in the study area.

## 1. Introduction

Typhoons are considered extremely devastating natural hazards worldwide, which have caused enormous property and human losses and severely impacted public health [1]. Under the influence of global warming and the rise in sea level [2], the frequency and intensity of natural disasters such as typhoons, rainstorms, and storm surges have increased. Furthermore, there has been an increase in the damage caused by these disasters. Affected by the same meteorological systems, typhoons, rainfall, and storm surges are three correlated variables. Based on previous reports [3,4,5,6], although the correlation between rainfall and storm surges is often weak, it has a significant impact on coastal urban flood management. Strong typhoons often bring on heavy rainfall and high surges. Floods can easily occur in coastal cities when typhoons occur as they often bring heavy rainfall and storm surges. For example, Typhoon Rammasun, with a maximum wind speed of 60 m/s, attacked Haikou in Hainan Province, China, from 17 July to 19 July 2014, resulting in heavy daily rainfall (509.2 mm) and high storm surges (3.83 m) on 18 July. The return period of both rainfall and storm surges is more than 100 years. This resulted in severe waterlog disasters in Haikou due to the joint impact of heavy rainfall and high tide blocking, which caused the deaths of eight people and losses worth nearly 1.4 billion dollars. Consequently, it is necessary to investigate the dependence and encountering probability of typhoons, rainfall, and storm surges.

At present, the dependence between bivariate variables of typhoons, rainfall, and storm surges has been analyzed. Zheng [3,4] employed a bivariate logistic threshold–excess model to quantify the dependence between extreme rainfall and storm surges. Lian [5] and Xu [7] investigated the joint probability of rainfall and storm surges using copula-based models in Fuzhou City, China. Hurk [8] used an ensemble of regional climate model simulations to demonstrate that the combined occurrence of heavy precipitation and storm surges is physically related in the Netherlands. Wu [9] and Dong [10] analyzed the joint return probability of typhoon wind speed and rainfall intensity in the typhoon-affected sea area. Konrad [11] found that roughly a third or more of the small and medium precipitation events in the southeastern and northeastern regions of the Eastern United States were connected to tropical cyclones. Matyas [12] investigated relationships between typhoon rainfall distribution, typhoon size, and the environment surrounding a typhoon and found that the radius of the outermost closed isobar (ROCI) can be a useful delineation of regions that receive typhoon rainfall. Zhu [13] estimated typhoon rainfall risk by Emanuel’s synthetic approach in Texas, and Lonfat [14] showed how rainfall rates decrease away from the typhoon center by using the Tropical Rainfall Measuring Mission (TRMM) microwave imager. Wang [15] selected an optimal copula function to develop a joint probability distribution function of storm surges and typhoon wind speeds. If only the joint characteristics of univariate or bivariate functions are analyzed, the factual flood mechanism in coastal zones cannot be explored. However, until now, there has been a lack of knowledge about multivariate joint probability distribution of typhoons, rainfall, and storm surges. The joint probability distribution can reveal the occurrence probability of multiple variables. Therefore, it is meaningful to investigate the trivariate joint probability distribution of typhoons, rainfall, and storm surges for flood management in coastal zones. The copula is an efficient tool to obtain a suitable multivariable distribution due to flexible selection of the marginal distribution. Recently, copula functions are increasingly being used in multivariate hydrologic event analysis. For instance, they have been used for flood frequency analysis [16,17,18,19,20], rainfall frequency analysis [21,22,23,24,25], and drought frequency analysis [26,27,28,29,30]. Such studies demonstrate that copulas are robust tools for the probabilistic analysis of hydrological data [27]. Therefore, copula functions are used to establish the trivariate joint probability distribution of typhoons, rainfall, and storm surges.

Expected annual damage (EAD) is used as a flood risk model based on the probability function and flood damage function [31]. Flood damage can be calculated by unit flood damage and the urban flood inundation model [32]. The Personal Computer Storm Water Management Model (PCSWMM) [33,34,35,36] is one urban flood inundation model that combines Geographic Information System (GIS) and the US Environmental Protection Agency (EPA) SWMM 5 [37] and can provide a complete package for one-dimensional (1D) and two-dimensional (2D) analysis of rainfall runoff processes for storm-water modeling in urban and rural areas. It has been applied in many areas [33,34,35,36].

In this study, the joint risk of rainfall and storm surges during typhoons is investigated by integrating principal component analysis, copula function, urban flood inundation model, and flood risk model methods. The main objectives of this study are to find a good statistical description of multivariate joint probability distribution of typhoon–rainfall–storm surge and then to evaluate the impact of typhoons on the joint risk of rainfall and storm surges. This study would provide a theoretical basis for flood risk management in coastal zones and a reference for the investigation of urban floods caused by multiple hazard factors. The remainder of the paper is organized as follows. The study area and data are described in the next section. Section 3 describes methods used in this study. In Section 4, the construction of the trivariate joint probability distribution of typhoons, rainfall, and storm surges with copula functions is presented. Meanwhile, the copula-based probability of rainfall and storm surges under typhoons and the impact of typhoons on flood risk caused by rainfall and storm surges are discussed. Finally, conclusions are given in Section 5.

## 2. Study Area and Data

**Figure 1:**
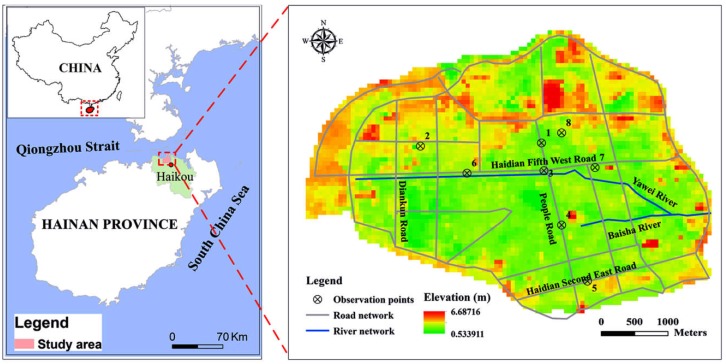
Location of the study area.

Haidian Island is located in the northern part of Haikou in Hainan Province, China, and is adjacent to the Qiongzhou strait (Figure 1). Because of the special location and low elevation of the Haidian Island, it is vulnerable to the joint impact of heavy rainfall and high storm surges. Furthermore, Haidian Island is one of the areas most seriously and frequently affected by typhoons in China. A total of 255 typhoons affected Haidian Island during 1951–2011, with an average of 4.2 typhoons per year. Heavy rainfall and high storm surges caused by typhoons have often resulted in severe flood damage to the island.

In this study, daily rainfall data and daily maximum storm surge data during 1974–2012 were collected from the Haikou hydrological station, which are provided by the Haikou Municipal Water Authority. The continuity of the data was checked. The typhoon data are available from the best-track dataset by the Shanghai Typhoon Institute of China Meteorological Administration (CMA). The dataset contains information on each typhoon track every 6 h, including the time, location (latitude and longitude), maximum sustained wind (MSW), and minimum pressure near the typhoon center. The CMA typhoon best-track dataset is included in the International Best Track Archive for Climate Stewardship (IBTrACS) project [38], which is an official World Meteorological Organization (WMO) global archiving and distribution resource for typhoon best-track data [39].

**Figure 2:**
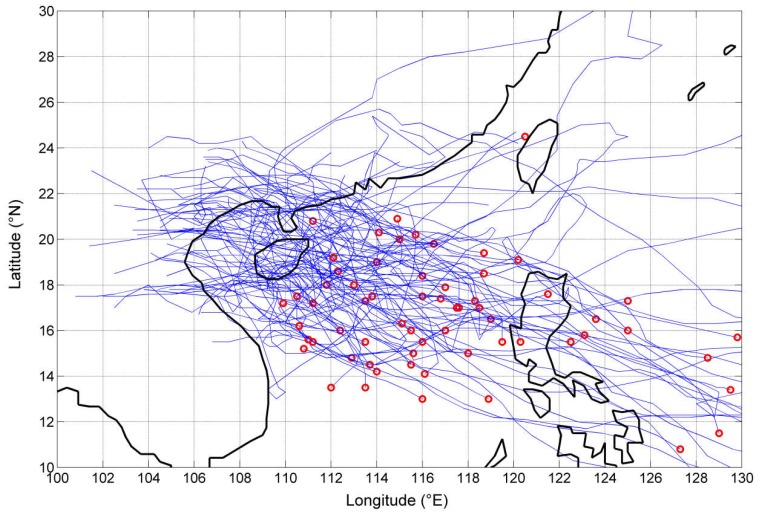
Typhoon tracks affecting Haidian Island. Red circles represent the origin points of typhoon events.

**Figure 3:**
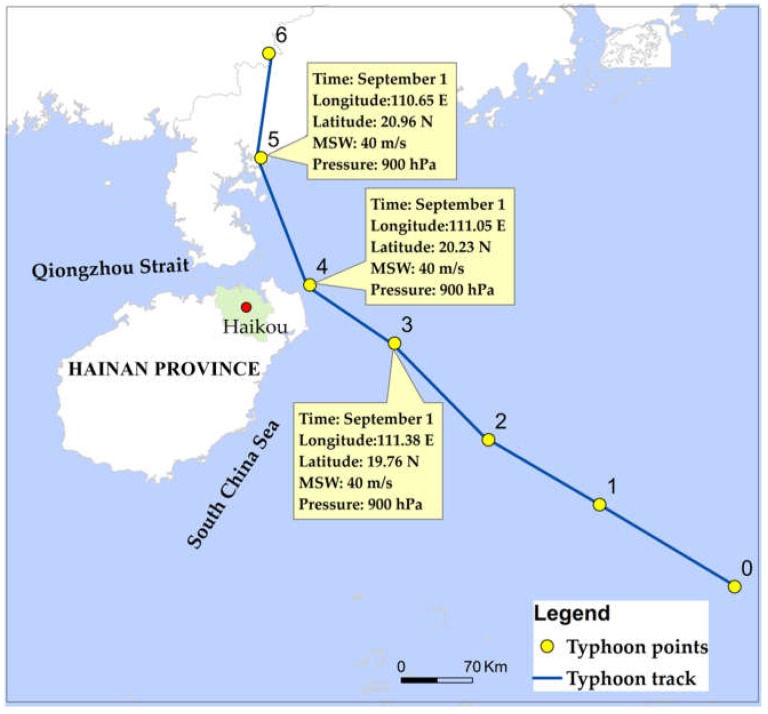
An example of a typhoon track affecting Haikou.

When the distance between the typhoon center and the study area is less than 500 km [40,41], the typhoon is considered to have potential impact on the study area. There were 128 typhoon events affecting Haidian Island from 1974 to 2012, and the tracks of those typhoon events are presented in Figure 2. As shown in the figure, most of the typhoons that affect Hainan Island originate in the South China Sea and approach the island from east to west. The data of each typhoon point include MSW, center pressure, and distance between the typhoon center and the study area. They were selected by the following steps. First, we selected the affected daily rainfall and storm surges by typhoons through the manual identification method. For example, on 1 September, daily rainfall and storm surges were affected by typhoons. Then, we determined the corresponding typhoon points (usually more than one) on 1 September and calculated the distance between typhoon points and Haikou. Finally, the typhoon point with the smallest distance was selected as the basic typhoon data on 1 September, since rainfall and storm surges are mainly affected by the typhoon position. As shown in Figure 3, typhoon points 3–5 affected Haikou on 1 September, and typhoon point 4 had the smallest distance. Therefore, the MSW, center pressure, and typhoon position of typhoon point 4 were selected as the basic typhoon data.

## 3. Methods

**Figure 4:**
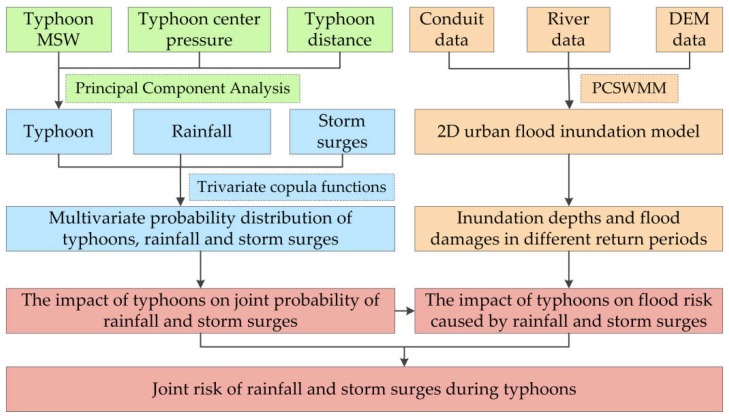
The overall framework. MSW: maximum sustained wind; DEM: digital elevation model; PCSWMM: Personal Computer Storm Water Management Model.

The overall framework of the study is shown in Figure 4. First, to quantify typhoons, principal component analysis was used to characterize typhoons by integrating MSW, center pressure, and distance between the typhoon center and the study area. Then, a multivariate joint probability distribution of typhoons, rainfall, and storm surges was constructed by trivariate copula functions. Third, the impact of typhoons on flood risk caused by rainfall and storm surges was evaluated based on an urban flood inundation model and a flood risk model. Finally, the joint risk of rainfall and storm surges during typhoons was estimated from two aspects: (1) the impact of typhoons on joint probability of rainfall and storm surges, and (2) the impact of typhoons on flood risk caused by rainfall and storm surges.

### 3.1. Principal Component Analysis

The existing literature has focused on the dependence between typhoons, rainfall, and storm surges used to characterize typhoons by MSW [9,10,11]. However, typhoons have many indices, such as MSW, center pressure, distance between typhoon points and the study area, SiR34 (the radius, in km, of 34 kt, i.e., tropical cyclone (TC) size), and so on. MSW cannot fully reflect the impact of typhoons on rainfall and storm surges. In this work, to analyze the multivariate joint probability distribution of typhoons, rainfall, and storm surges, typhoons were quantified by a synthetic parameter integrating MSW, center pressure, and distance between the typhoon center and the study area, since these variables all have significant dependence with rainfall and storm surges (Table 1). As shown in Table 1, since SiR34 has a lower correlation with rainfall and storm surges, it was not adopted in the base parameter set to perform the principal component analysis. Therefore, in this study, principal component analysis was used to characterize typhoons by integrating MSW, center pressure, and distance between the typhoon center and the study area.

Principal component analysis is one of the most widely applied tools for simplifying a dataset by reducing multidimensional datasets to a smaller number of dimensions than the original representation without changing the characteristics of the original data [42]. For an *n*-dimensional observation sample matrix *S* with *p* eigenvalues, the principal component can be calculated with the following steps:

Step 1. Establish observation sample matrix *S*:

(1) $$S=\left[\begin{array}{cccc} s_{11} & s_{12} & \cdots & s_{1p}\\ s_{21} & s_{22} & \cdots & s_{2p}\\ & & \vdots & \\ s_{n1} & s_{n2} & \cdots & s_{np} \end{array}\right]$$

Step 2. Convert sample matrix *S* to normalized matrix *Y*:

1. Normalized matrix for positive indices:

(2) $$y_{ij}=\frac{s_{ij}-\min(s_{ij})}{\max(s_{ij})-\min(s_{ij})}$$

2. Normalized matrix for negative indices:

(3) $$y_{ij}=\frac{\max(s_{ij})-s_{ij}}{\max(s_{ij})-\min(s_{ij})}$$

Step 3. Calculate the correlation coefficient matrix of the normalized matrix:

(4) $$R=\left[\begin{array}{cccc} r_{11} & r_{12} & \cdots & r_{1p}\\ r_{21} & r_{22} & \cdots & r_{2p}\\ & & \vdots & \\ r_{p1} & r_{p2} & \cdots & r_{pp} \end{array}\right]$$

Step 4. Calculate eigenvalues *λ* of correlation coefficient matrix *R*:

(5) |R−λI|=0

where *I* is the *p* by *p* identity matrix, |·| is the determinant operator, and λ represents the *p* eigenvalues ranked in decreasing order, $\lambda_{1}\geq \lambda_{2}\geq \cdots \geq \lambda_{p}\geq 0$.

Step 5. Choose principal components $U_{1},U_{2},\cdots ,U_{m}$.

When the variance cumulative contribution rate of *m* principal components $\sum_{i=1}^{m}\lambda_{i}/\sum_{i=1}^{p}\lambda_{i}$ is close to 1 (generally greater than 85%), the factor variables $U_{1},U_{2},\cdots ,U_{m}$ are known as the first, second, ..., *m*th principal components of the original variables, respectively, which are expressed as:

(6) $$U_{j}=b_{j}^{T}y$$

where *y* is the normalized matrix mentioned in Equations (2) and (3), and $b_{j}$ is the eigenvector of eigenvalue $\lambda_{j}$. It can be calculated by the following formula.

(7) $$Rb=\lambda_{j}^{}b$$

Step 6. Characterize samples by integrating principal components:

The sample values can be integrated by the following formulas [43,44,45].

(8) $$F=\sum_{j=1}^{m}\omega_{j}U_{j}$$

(9) $$\omega_{j}=\frac{\lambda_{j}}{\sum_{i=1}^{m}\lambda_{i}}\;j=1,\;2,\ldots ,\;m$$

where $\omega_{j}$ is the weighting coefficient of principal component $U_{j}$, and $\lambda_{j}$ is the variance contribution rate of principal component $U_{j}$.

### 3.2. Copula Function

$F_{1},F_{2},\cdots ,F_{n}$ are marginal distributions of $X_{1},X_{2},\cdots ,X_{n}$, respectively. According to Sklar’s theorem [46], if $F_{1},F_{2},\cdots ,F_{n}$ are continuous, there exists an n-copula C as follows:

(10) $$F(x_{1},x_{2},\cdots ,x_{n})=C(F_{1}(x_{1}),F_{2}(x_{2}),\cdots ,F_{n}(x_{n}))$$

In this study, two elliptical copulas (Gaussian copula and Student’s t copula) and three Archimedean copulas (Gumbel copula, Clayton copula, and Frank copula) were employed to build joint distributions of typhoon–rainfall, typhoon–storm surge, and rainfall–storm surge. Trivariate Gaussian copula, Gumbel copula, and Student’s t copula were employed to construct the joint distribution of typhoons, rainfall, and storm surges. The above copula functions are presented in Appendix A.

The joint probability, co-occurrence probability, and conditional probability of typhoons, rainfall, and storm surges can be calculated by copulas. For *T* representing the typhoon, *H* representing rainfall, and *Z* representing the storm surge, the joint probability of at least one variable (typhoon *T*, rainfall *H*, or storm surge *Z*) exceeding its extreme values is denoted as P∪(t,h,z). The expression of P∪(t,h,z) is as follows [47,48]:

(11) P∪(t,h,z)=P((T>t)∪(H>h)∪(Z>z))=1−F(t,h,z)

The co-occurrence probability of *T*, *H*, and *Z* all exceeding certain extremes is denoted as P∩(t,h,z). The expression of P∩(t,h,z) is as follows [47,48]:

(12) $$\begin{array}{cc} P\cap (t,h,z) & =P\left(\left(T>t)\cap (H>h)\cap (Z>z\right)\right)\\ & =1-F_{T}(t)-F_{H}(h)-F_{Z}(z)+F(t,h)+F(t,z)+F(h,z)-F(t,h,z) \end{array}$$

The conditional probability that *H* and *Z* exceed a certain extreme when *T* has exceeded the extreme is denoted as P(H>h,Z>z|T>t). The expression of P(H>h,Z>z|T>t) is as follows:

(13) $$\begin{array}{cc} P(h,z|t) & =P(H>h,Z>z|T>t)\\ & =\frac{1-F_{T}(t)-F_{H}(h)-F_{Z}(z)+F(t,h)+F(t,z)+F(h,z)-F(t,h,z)}{1-F_{T}(t)} \end{array}$$

### 3.3. Expected Annual Damage Evaluation

In this study, flood risk is defined as the product of probability and damages [49]. The expected annual damage (EAD) is then found to express the flood risk, which can be calculated by integrating the flood damage function with the probability function. An approximation for calculating EAD is described in [31]:

(14) $$\mathrm{EAD}=\sum_{i=1}^{i=m}D_{i}\times \Delta P_{i}$$

(15) $$D_{i}=\frac{D_{P_{i-1}}+D_{P_{i}}}{2}$$

where *D_P_* is the damage caused by a flood of exceedance probability *P*, *m* is the number of probability increments, and *D_i_* is the average flood damage (mean of *D_pi_* and *D*_*pi*−1_) during probability increment $\Delta P_{i}$ for the *i*th interval. Flood damages are calculated by unit flood damage and maximum inundation depth. The inundation depth is calculated by PCSWMM, which is introduced in Appendix B.

## 4. Results and Discussion

### 4.1. Quantification of Typhoons

In order to establish the multivariate joint probability of typhoons, rainfall, and storm surges, typhoons should be quantified first. To quantify typhoons, characterization by principal component analysis was performed, integrating MSW, center pressure, and distance between the typhoon center and the study area. The results reveal that, on the basis of eigenvector loadings, the first principal component (PC1) with an eigenvalue of 0.093 is able to explain 59.1% of the total variation, whereas the second principal component (PC2) with an eigenvalue of 0.062 explains 39.3% of the variation, and both PC1 and PC2 explain 98.4% of the total variation (Table 2). Therefore, the first two principal components are sufficient to replace the original data information.

From the score coefficient matrix in Table 3, the score coefficients of MSW, center pressure, and distance in PC1 are 0.745, 0.589, and 0.312, respectively. MSW and center pressure have higher score coefficients than distance, indicating that PC1 is the comprehensive reaction of MSW and center pressure. The second PC, negatively loaded with MSW (−0.247) and center pressure (−0.19), is positively loaded with the distance between the typhoon center and the study area (0.95). The proportion of distance is the highest in PC2, which indicates that PC2 is a description of distance. Therefore, PC1 and PC2 can fully reflect MSW, center pressure, and the distance, indicating that the principal component analysis results are reasonable. Furthermore, the principal components can be described as follows.

(16) $$U_{1}=0.74534\;S_{1}+0.589417\;S_{2}+0.311539\;S_{3}$$

(17) $$U_{2}=-0.24722\;S_{1}-0.18963\;S_{2}+0.950223\;S_{3}$$

where $S_{1}$ is MSW, $S_{2}$ is center pressure, and $S_{3}$ denotes the distance between the typhoon center and the study area.

From Equations (8) and (9), typhoon *T* can be quantified by the following equation [43,44,45]:

(18) $$F=0.6\;U_{1}+0.4\;U_{2}$$

### 4.2. Multivariate Joint Probability Distribution of Typhoons, Rainfall, and Storm Surges

#### 4.2.1. Correlation among Typhoons, Rainfall, and Storm Surges

The quantitative correlation among variables was analyzed using Pearson’s correlation coefficient *r* and two nonparametric dependence measures, Kendall’s τ and Spearman’s ρ. Table 4 presents corresponding correlation measures between typhoons, rainfall, and storm surges. The correlation between variables was found to be statistically significant at 1% significance level, as checked by a standard two-tailed *t*-test. The correlation is largest between typhoons and rainfall (Spearman’s ρ = 0.368 at 1% significance level), while it is the smallest between rainfall and storm surges (Kendall’s τ = 0.142 at 1% significance level). The reason may be that rainfall and storm surges are affected by various weather systems, and typhoon is only one factor. Additionally, the angle of approach of a typhoon may also cause the low correlation between rainfall and storm surges (e.g., being on the left side of the typhoon can reduce the storm surge, while it produces maximum rainfall on the left side). Even though the correlation between variables is low, it can have significant implications for flood risk estimates, and there are still many studies about their joint probability analysis [3,4,5,6,7,8]. Furthermore, to analyze the correlation between extreme events, upper-tail correlation coefficient λ_u_ was also evaluated, and the introduction of λ_u_ is described in the literature in detail [50]. As shown in Table 4, upper-tail correlation coefficients are higher than Pearson’s correlation coefficients, Kendall’s τ, and Spearman’s ρ among typhoons, rainfall, and storm surges, indicating that the correlation among extreme events is stronger.

#### 4.2.2. Trivariate Joint Probability Distribution of Typhoon–Rainfall–Storm Surge

**Figure 5:**
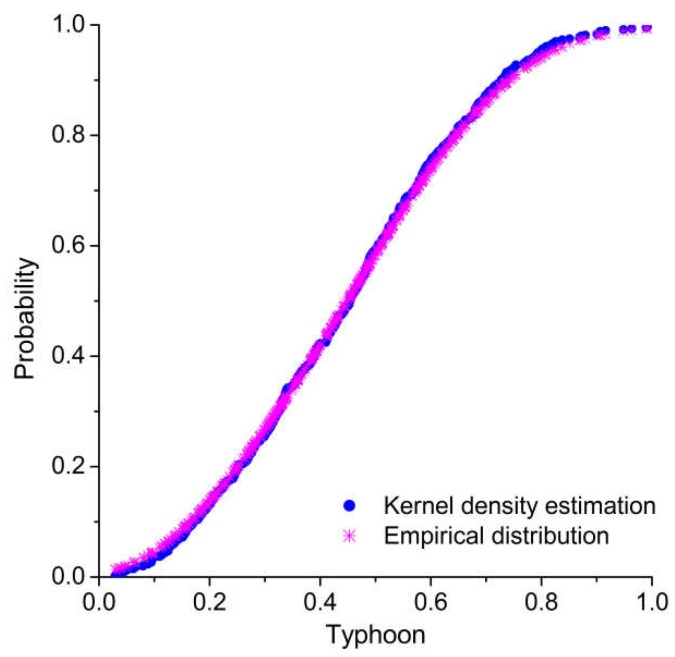
Cumulative probability distribution of typhoon.

**Figure 6:**
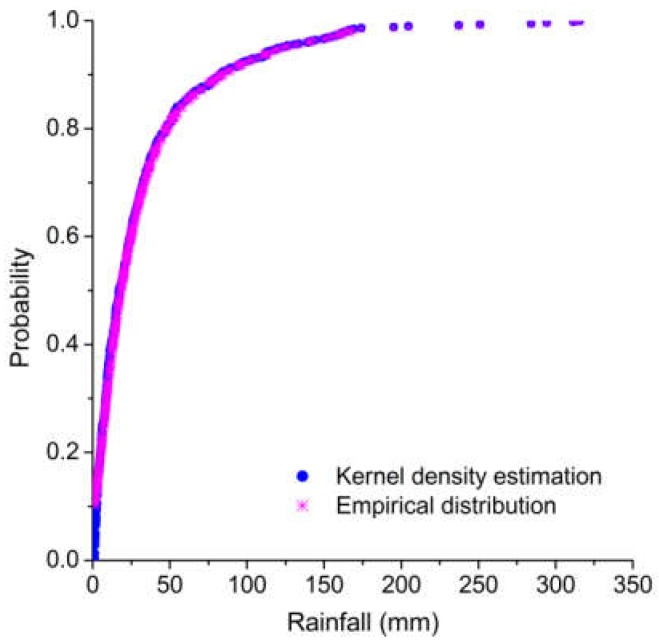
Cumulative probability distribution of rainfall.

**Figure 7:**
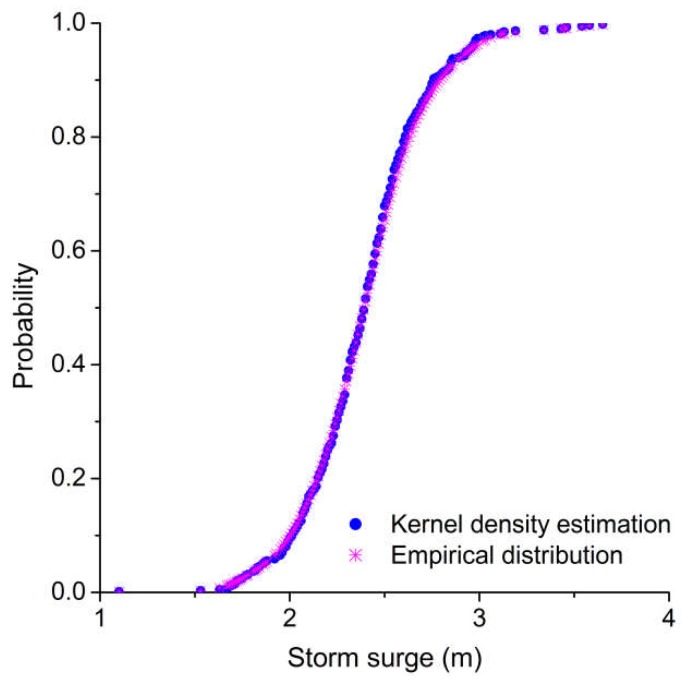
Cumulative probability distribution of storm surge.

**Figure 8:**
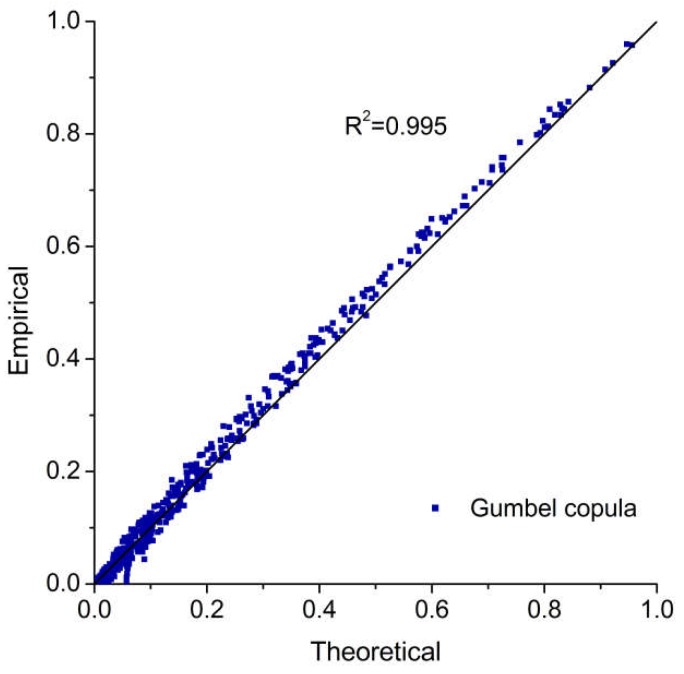
P–P plot of the joint distribution of typhoons, rainfall, and storm surges.

**Figure 9:**
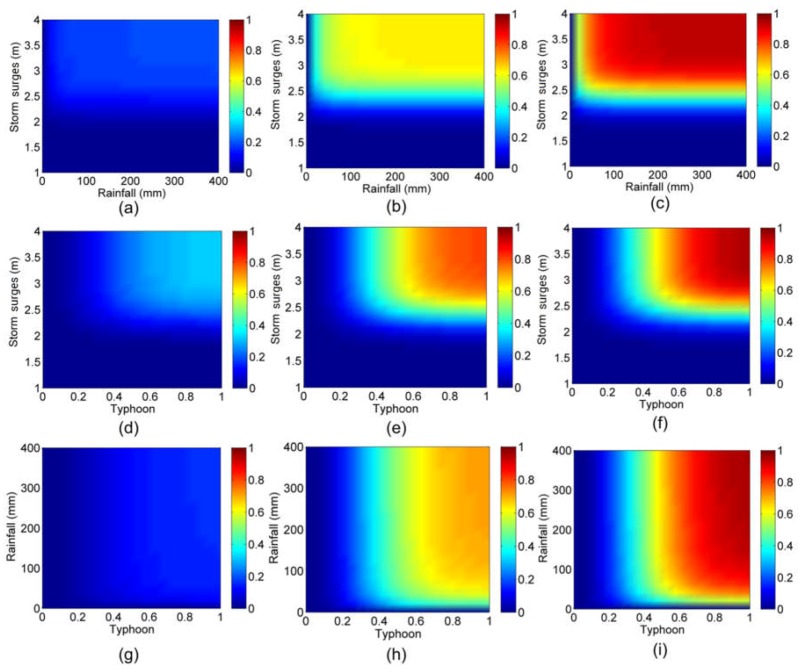
Some bivariate views of the trivariate probability distribution of typhoons, rainfall, and storm surges. Joint probability distribution of rainfall and storm surges when typhoon value is (**a**) 0.2, (**b**) 0.5, (**c**) 0.8; joint probability distribution of typhoons and storm surges when rainfall is (**d**) 10 mm, (**e**) 50 mm, (**f**) 150 mm; joint probability distribution of typhoons and rainfall when storm surge is (**g**) 2.0 m, (**h**) 2.5 m, (**i**) 3.0 m.

The nonparametric kernel density estimation [51,52] was used to establish the marginal distribution of typhoons, rainfall, and storm surges. As shown in Table 5, all of the computed values of the Kolmogorov–Smirnov (K–S) statistic *D* are lower than the critical values (*D*_0.01_ = 0.067). Furthermore, a comparison of kernel density estimations and empirical distributions of typhoons, rainfall, and storm surges are presented in Figure 5, Figure 6 and Figure 7, indicating that the kernel density estimation can properly estimate the distribution functions of typhoons, rainfall, and storm surges. As for bivariate joint distributions, typhoon–rainfall, typhoon–storm surge, and rainfall–storm surge are all best fitted by the Gumbel copula due to minimal Akaike information criterion (AIC) statistics being found for these bivariate distributions (Table 6). Table 7 shows the results of goodness of fit of the trivariate distribution of typhoon–rainfall–storm surge. It can be seen that typhoon–rainfall–storm surge is best fitted by the Gumbel copula, as it has the lowest AIC statistics and passes the K–S test. Figure 8 illustrates the probability–probability (P–P) plot of the joint distribution of typhoon–rainfall–storm surge. The coefficient of determination between empirical distribution and copula distribution is above 0.99, which indicates that the selected copula distribution is reasonable, and the selected parameters are adoptable. The trivariate joint probability distribution of typhoons, rainfall, and storm surges is expressed in Figure 9. With an increase in typhoons, rainfall, and storm surges, their joint probability distribution increases.

#### 4.2.3. Joint Probability of Typhoon–Rainfall–Storm Surge Analysis

The joint probability and co-occurrence probability of typhoons, rainfall, and storm surges are calculated from Equations (11) and (12). From Table 8, we can conclude the following: (1) With a decrease in the return period (RP), the joint probability of typhoons, rainfall, and storm surges increases. (2) The trivariate joint probability is always greater than the co-occurrence probability in all conditions. Hence, the simultaneous occurrence of typhoons, rainfall, and storm surges all exceeding certain threshold values is less frequent. However, one variable (typhoon, rainfall, or storm surge) more frequently exceeds its threshold value. (3) The joint probability P^∪^ is nearly two times the univariate occurrence probability of typhoons, rainfall, and storm surges, and flooding would easily occur when either of them exceeds the threshold. However, the flood design standard in China is only determined by univariate analysis (i.e., rainfall frequency analysis), which would highly underestimate the flood risk in coastal regions.

Table 8 also shows a comparison of flood risk with and without considering the dependence between typhoons, rainfall, and storm surges. From Table 8, we can conclude that P∩(t,h,z) is much higher than $P^{*}\cap (t,h,z)$ in different return periods. Here, P∩(t,h,z) denotes the co-occurrence probability that typhoon *T*, rainfall *H*, and storm surge *Z* all exceed a certain magnitude. $P^{*}\cap (t,h,z)$ denotes P((T>t)∩(H>h)∩(Z>z)) without considering the dependence between these flood drivers. This indicates that ignoring the dependence between these flood drivers may inappropriately characterize the flood risk in coastal regions and can lead to underestimating it. These findings are in agreement with the results found by Salvadori et al. [53].

### 4.3. Joint Risk of Rainfall and Storm Surges during Typhoons on Haidian Island

#### 4.3.1. Impact of Typhoons on Joint Probability of Rainfall and Storm Surges

**Figure 10:**
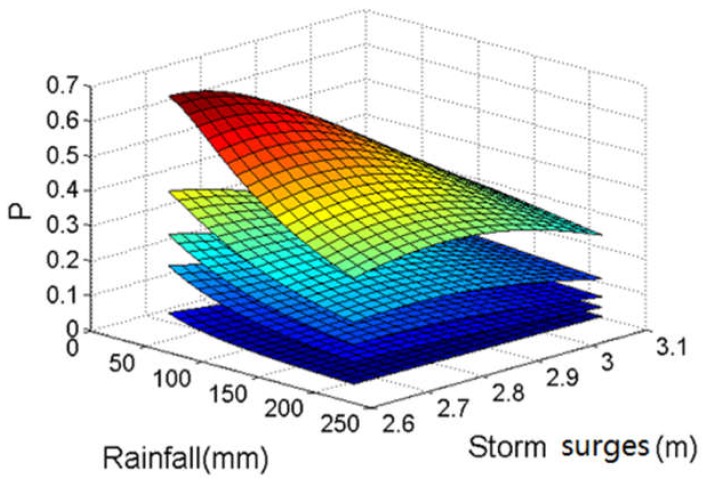
Co-occurrence probability of rainfall and storm surges under different return period (RP) typhoon conditions (from top to bottom: 50-year RP typhoon, 20-year RP typhoon, 10-year RP typhoon, 5-year RP typhoon, no typhoon).

Figure 10 and Table 9 show the co-occurrence probability of rainfall and storm surges under different typhoon RP conditions. The co-occurrence probability of rainfall and storm surges increases by at least 200% when a typhoon occurs (see Table 9). Furthermore, with an increase in typhoon RP, the conditional probability of rainfall and storm surges increases rapidly. For example, the co-occurrence probability of a 50-year RP rainfall and 50-year RP storm surge is only 0.004 when there is no typhoon, and it increases to 0.036 under 5-year RP typhoon conditions. When a 50-year RP typhoon occurs, it increases to 0.225. Furthermore, the probabilities P(H>h) and P(Z>z) significantly increase when a typhoon has occurred (see Table 9). Therefore, the coastal urban flood management strategy should pay more attention to the joint impact of rainfall and storm surges on flood risk when a typhoon has occurred.

#### 4.3.2. Impact of Typhoons on Flood Risk Caused by Rainfall and Storm Surges

The impact of typhoons on flood risk caused by rainfall and storm surges was estimated based on an urban flood inundation model and an expected annual damage model. In this study, expected annual damage (EAD) was then found to express the flood risk, which can be calculated by integrating the flood damage function with the probability function. Flood extent and depth are the most important indicators of flood damage, often denoted as depth-damage curves [54]. However, a credible regional depth-damage curve is difficult to obtain due to the complexity of urban contexts [55]. Therefore, we used unit flood damage and inundation depth for cost estimation [32]. The unit flood damage on Haidian Island is from Lian et al. [32], which was selected as the average economic loss in unit inundation depth of the four rainfall events, RE-1 (12 October 2008), RE-2 (5 August 2009), RE-3 (28 September 2009), and RE-4 (12 October 2009). The inundation depth and economic loss in the four rainfall events are shown in Table 10.

**Figure 11:**
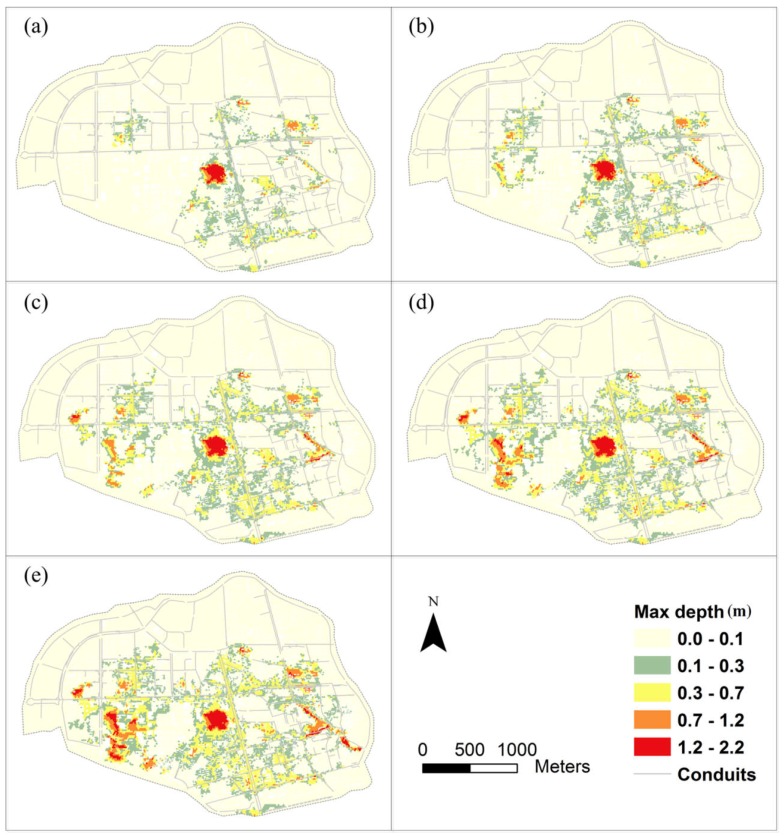
Maximum inundation depths in different RPs of rainfall and storm surges: (**a**) 5-year RP, (**b**) 10-year RP, (**c**) 20-year RP, (**d**) 50-year RP, (**e**) 100-year RP.

Inundation depth was calculated by PCSWMM. The calculation steps of inundation depth are introduced in detail in Appendix B. The maximum inundation depths in different RPs of rainfall and storm surges are presented in Figure 11. Flood damage was calculated by maximum inundation depths and unit flood damage. Table 11 shows that flood damage increases quickly with the increase of return period, and P(*H,Z|T*) is much more than P(*H,Z*). After calculation, EAD is 0.82 million dollars when there is no typhoon, and it rises to 3.27 million dollars when a typhoon has occurred. This indicates that typhoons greatly increase the flood risk in coastal zones. The main reason is the increase of the occurrence probability of rainfall and storm surges in typhoon conditions.

## 5. Conclusions

In this study, the joint risk of rainfall and storm surges during typhoons was investigated by integrating principal component analysis, copula-based probability analysis, urban flood inundation model, and flood risk model methods. First, principal component analysis was used to quantify typhoons by integrating MSW, center pressure, and distance between the typhoon center and the study area. Following this, the Gumbel copula was found to be a robust and proper function for the joint probability of typhoons, rainfall, and storm surges. Then, the joint probability, co-occurrence probability, and conditional probability were indicated by the Gumbel copula. The results of joint probability indicate that ignoring the dependence between these flood drivers may inappropriately characterize the flood risk in coastal regions and can lead to underestimating it. For conditional probability, the co-occurrence probability of rainfall and storm surges increases by at least 200% during typhoons. Therefore, coastal urban flood management should pay more attention to the joint impact of rainfall and storm surges on flood risk when a typhoon has occurred. Furthermore, when a typhoon has occurred, The expected annual damage (EAD) increases from 0.82 million dollars to 3.27 million dollars. This indicates that typhoons greatly increase the flood risk in coastal cities.

These messages are useful for practical design and planning, since all flood hazards, such as typhoons, rainfall, and storm surges, are considered. However, the study also has certain limitations. First, since the main objective of this study is to evaluate the impact of typhoons on the joint risk of rainfall and storm surges, the impact of typhoon size and typhoon conditions on rainfall distribution was not taken into consideration and needs to be explored in our future work. Second, the limited socioeconomic data in the study area restricted monetizing flood damage. Referring to Lian et al. [32], flood damage was defined as a function of inundation depth and flood unit cost in this study. Future research work could focus on improving the accurate quantification of flood damage. Furthermore, the impact of the uncertainty of copulas on flood risk estimation is also an important research focus in our future work.

## Figures and Tables

**Table 1:** Pearson’s correlation coefficient between typhoon parameters and rainfall and storm surges.

Typhoon Parameters	Rainfall	Storm Surges
MSW	0.454 *	0.376 *
Center pressure	−0.448 *	−0.304 *
Distance between typhoon center and study area	−0.574 *	−0.354 *
SiR34	0.189	0.153

* Correlation is significant at 1% significance level; MSV: maximum sustained wind; SiR34: the radius, in km, of 34 kt, i.e., tropical cyclone (TC) size.

**Table 2:** Index eigenvalue and contribution rate.

Principal Component	Eigenvalue	Contribution Rate (%)	Cumulative Contribution Rate (%)
U_1_	0.092988	59.10052	59.10052
U_2_	0.06183	39.29707	98.39759
U_3_	0.002521	1.60241	100

**Table 3:** Matrix of component score coefficients.

Variable	U_1_	U_2_	U_2_
S_1_	0.74534	−0.24722	−0.61915
S_2_	0.589417	−0.18963	0.785257
S_3_	0.311539	0.950223	−0.00437

**Table 4:** Correlation among typhoons, rainfall, and storm surges.

Dependence Measure	Typhoon–Rainfall	Typhoon–Storm Surge	Rainfall–Storm Surge
Pearson’s *r*	0.339	0.202	0.262
Kendall’s τ	0.249	0.102	0.142
Spearman’s ρ	0.368	0.151	0.209
Upper-tail correlation coefficient λ_u_	0.416	0.218	0.300

Correlation is significant at 1% significance level.

**Table 5:** Fitting results of marginal distribution functions. K–S, Kolmogorov–Smirnov.

Variate	Typhoon	Rainfall	Storm Surge
K–S statistic D	0.017	0.066	0.011

**Table 6:** Fitting results of bivariate distribution functions. AIC, Akaike information criterion.

Pair	Gaussian Copula		Student’s t-Copula		Clayton Copula		Frank Copula		Gumbel Copula	
	KS	AIC	KS	AIC	KS	AIC	KS	AIC	KS	AIC
Typhoon–rainfall	0.081	−963.566	0.084	−948.301	0.084	−542.389	0.080	−978.488	**0.066**	**−1169.123**
Typhoon–storm surge	0.049	−1047.931	0.046	−1082.368	0.066	−726.048	0.051	−1030.418	**0.040**	**−1274.238**
Rainfall–storm surge	0.086	−815.687	0.085	−837.389	0.085	−570.130	0.085	−825.573	**0.064**	**−971.122**

The values of the distributions in bold were selected.

**Table 7:** Fitting results of trivariate distribution functions.

Distribution	Gaussian Copula	Student’s t Copula	Gumbel Copula
AIC	−925.276	−901.062	**−957.371**
K–S	0.068	0.069	**0.057**

The values of the distributions in bold were selected.

**Table 8:** Joint probability (P^∪^) and co-occurrence probability (P^∩^) of typhoons, rainfall, and storm surges.

Return Period (Years)	5	10	20	50	100
P∪(t,h,z)	0.430	0.233	0.121	0.050	0.025
P∩(t,h,z)	0.053	0.024	0.011	0.004	0.002
$P^{*}\cap (t,h,z)$	0.008	0.001	1.25 × 10^−4^	8 × 10^−6^	1 × 10^−6^

P∪(t,h,z) denotes P((T>t)∪(H>h)∪(Z>z)), P∩(t,h,z) denotes P((T>t)∩(H>h)∩(Z>z)), and $P^{*}\cap (t,h,z)$ denotes P((T>t)∩(H>h)∩(Z>z)) without considering the dependence between these flood drivers.

**Table 9:** Probabilities P(H>h|T>t), P(Z>z|T>t), P(H>h,Z>z), and P(H>h,Z>z|T>t).

RP (years)	Typhoon T	Rainfall H (mm)	Storm Surge Z (m)	P(*H|T*)	P(*Z|T*)	P(*H,Z*)	P(*H,Z|T*)
5	0.65	46	2.67	0.434	0.334	0.069	0.266
10	0.74	80	2.82	0.375	0.258	0.027	0.239
20	0.81	125.6	2.95	0.345	0.221	0.012	0.229
50	0.9	210.7	3.1	0.328	0.198	0.004	0.225
100	0.96	294.2	3.21	0.322	0.191	0.002	0.223

**Table 10:** Inundation depth and economic loss for different rainfall events [32].

Year	Rainfall Events	Inundation Depth (m)	Economic Loss (Million Dollars)
2008	RE-1	1.5	51.72
2009	RE-2	0.5	9.01
	RE-3	0.7	6.06
	RE-4	0.5	4.74

**Table 11:** P(*H,Z*), P(*H,Z|T*), and flood damages.

Return Period (Years)	5	10	20	50	100
P(*H,Z*)	0.069	0.027	0.012	0.004	0.002
P(*H,Z|T*)	0.266	0.239	0.229	0.225	0.223
Flood damage (million dollars)	7.38	11.17	16.51	20.66	24.83

**Figure A1:**
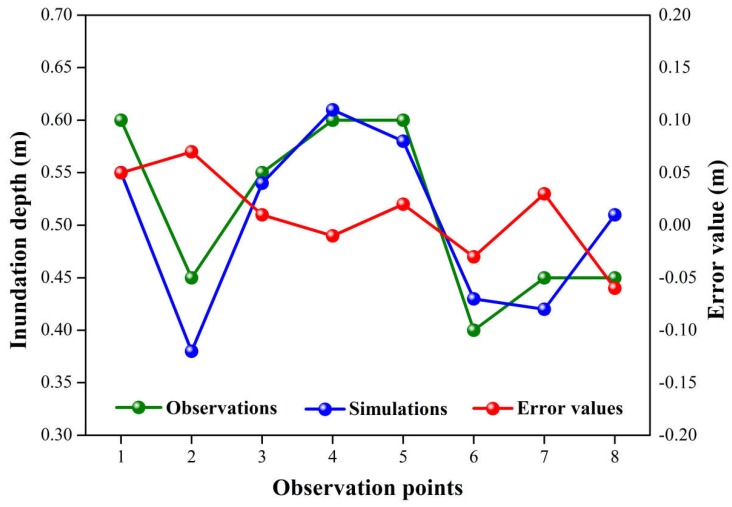
Calibration of the PCSWMM model.

## Appendix A. Introduction of Copula Functions

In this study, two elliptical copulas (Gaussian copula and Student’s t copula) and three Archimedean copulas (Gumbel copula, Clayton copula, and Frank copula) were employed to build joint distributions of typhoon–rainfall, typhoon–storm surge, and rainfall–storm surge. Common bivariate copula functions are presented in Table A1.

**Table A1:** Common bivariate copula functions.

Copulas	C(*u,v*)
Gumbel	$C(u,v)=\exp\{-{\left[{\left(-\ln u\right)}^{\theta }+{\left(-\ln v\right)}^{\theta }\right]}^{1/\theta }\}$
Clayton	$C(u,v)={\left(u^{-\theta }+v^{-\theta }-1\right)}^{-1/\theta }$
Frank	$C(u,v)=-\frac{1}{\theta }\ln[1+\frac{\left(e^{-\theta u}-1)(e^{-\theta v}-1\right)}{e^{-\theta }-1}]$
Gaussian	$C(u,v)=\int_{-\infty }^{\Phi^{-1}(u)}\int_{-\infty }^{\Phi^{-1}(v)}\frac{1}{2\pi \sqrt{1-\rho^{2}}}\exp\left(\frac{-(r^{2}+s^{2}-2\rho rs)}{2(1-\rho^{2})}\right)drds$
Student’s t	$C(u,v,\rho ,\nu )=\int_{-\infty }^{T_{\nu }{}^{-1}(u)}\int_{-\infty }^{T_{\nu }{}^{-1}(v)}\frac{1}{2\pi \sqrt{1-\rho^{2}}}\exp{\left(1+\frac{r^{2}+s^{2}-2\rho rs}{\nu {\left(1-\rho \right)}^{2}}\right)}^{-\frac{\nu +2}{2}}drds$

The trivariate Gaussian copula, Student’s t copula, and Gumbel copula were used to establish the joint distribution of typhoons, rainfall, and storm surges. Trivariate copula functions are as follows.

1. Trivariate Gaussian copula:

(A1) C(u1,u2,u3)=∫−∞Φ−1(u1)∫−∞Φ−1(u2)∫−∞Φ−1(u3)1(2π)32|∑|12exp(−12wT∑−1w)dw

where $\Phi^{-1}(\cdot )$ is the quintile function of a standard univariate normal function, $\sum =\left[\begin{array}{lll} 1 & \cdots & \rho_{1m}\\ \cdots & \cdots & \cdots \\ \rho_{m1} & \cdots & 1 \end{array}\right]$ is the correlation coefficient matrix with $\rho_{ij}=\left\{ \begin{array}{l} 1;i=j\\ \rho_{ji};i\neq j \end{array}\right.$, $-1\leq \rho_{ij}\leq 1$, *m* is the variable dimensions, and $w={\left[w_{1},w_{2},\cdots ,w_{m}\right]}^{T}$ is the integrand variable matrix.

2. Trivariate Student’s t-copula:

(A2) C(u1,u2,u3)=∫−∞Tv−1(u1)∫−∞Tv−1(u2)∫−∞Tv−1(u3)Γ(v+32)Γ(v2)1(πv)32|∑|12(1+wT∑−1wv)−(v+32)dw

where $T_{v}{}^{-1}(\cdot )$ is the quintile function of Student’s t distribution function with *v* degrees of freedom, $\sum =\left[\begin{array}{lll} 1 & \cdots & \rho_{1m}\\ \cdots & \cdots & \cdots \\ \rho_{m1} & \cdots & 1 \end{array}\right]$ is the correlation coefficient matrix with $\rho_{ij}=\left\{ \begin{array}{l} 1;i=j\\ \rho_{ji};i\neq j \end{array}\right.$, $-1\leq \rho_{ij}\leq 1$, *m* is the variable dimensions, and $w={\left[w_{1},w_{2},\cdots ,w_{m}\right]}^{T}$ is the integrand variable matrix.

3. Trivariate Gumbel copula:

(A3) $$C(u_{1},u_{2},u_{3})=\exp(-{\left({\left(-\ln u_{1}\right)}^{\theta }+{\left(-\ln u_{2}\right)}^{\theta }+{\left(-\ln u_{3}\right)}^{\theta }\right)}^{1/\theta })$$

where *θ* is a parameter of the copula function.

The parameters of the above distributions were estimated by the maximum likelihood method. The Kolmogorov–Smirnov (K–S) test was used to assess goodness of fit. The best-fit copulas were selected by the Akaike information criterion (AIC).

The Kolmogorov–Smirnov statistic D is defined as follows:

(A4) $$D=\underset{1\leq k\leq n}{\max}\{\left|C_{k}-\frac{i}{n}\right|,\left|C_{k}-\frac{i-1}{n}\right|\}$$

where $C_{k}$ is the theoretical distribution of the measured samples, *i* is the serial number of the measured samples in ascending order, and *n* is the number of samples. When the statistic D is less than the critical value $D_{\alpha }$, the test is accepted.

The formula of AIC is defined as follows:

(A5) AIC=2Nln(RMSE)+2k

where N is the number of samples, RMSE is the root-mean-square error of samples, and k is the number of variables. A smaller AIC value indicates a better fit.

## Appendix B. Urban Flood Inundation Model on Haidian Island

PCSWMM, developed by CHI, Canada, is widely applied in the simulation of surface hydrological processes and drainage network flows [33,34,35,36]. Unlike SWMM, which can only simulate 1D conduit flow and river flow, PCSWMM can accurately simulate unsteady 2D surface flow above ground through a 2D floodplain model. In this study, the urban flood inundation model on Haidian Island was established by PCSWMM. The calculation steps of inundation depth are as follows: (1) Prepare data. The basic data of the urban flood inundation model include digital elevation model (DEM) data, river data (e.g., river cross-section and distance between cross-sections), drainage data (e.g., junction depth, conduit size, etc.), and obstruction data (i.e., building data). DEM data (Figure 1) were accessed from the Institute of Geographic Sciences and Natural Resources Research, Chinese Academy of Sciences (http://www.resdc.cn/Default.aspx), and the DEM resolution is 25 m. River data and drainage data were provided by the Haikou Municipal Water Authority. The obstruction data were extracted from satellite remote sensing images through Environment for Visualizing Images (ENVI) software. (2) Establish 1D model. The dynamic wave method was used to calculate hydraulic simulations of drainage conduits. Based on the specific circumstances of the study area, for example, the study area is a small catchment and there is not enough soil data; the Horton model was used to calculate infiltration, since it is suitable for small catchments and areas where soil data are lacking. The parameters of the PCSWMM model were calibrated by trial and error and the recommended ranges from relevant references [37,56]. (3) Establish 2D model. The size of the 2D grid is 25 m × 25 m, and the study area was divided into 23,578 grids. The 1D conduit model and 2D floodplain model were integrated by the orifice connection method [57]. (4) Calibrate urban flood inundation model. Calibration data for storm events was based on actual flood inundation data during the Rammasun typhoon in July 2014, which was obtained through field investigation. Figure A1 shows observations and calculation inundation depths in different points of the study area. The observation points are indicated in Figure 1. The error values between observations and calculations are smaller than 0.1 m, which indicates that the model is reliable.
